# Innovation Elements, Incubation Capacity, and Incubation Performance in Biomedical Incubation Platforms: Moderating Role of Customized Services

**DOI:** 10.3389/fpubh.2022.873875

**Published:** 2022-05-11

**Authors:** Qian Jiang, Dan Wang, Yunfeng Wang, Bingye Wu

**Affiliations:** Business School, University of Sanya, Sanya, China

**Keywords:** biomedical incubation platform, innovation elements, incubation capacity, customized service, incubation performance

## Abstract

Biomedical incubation platforms make full use of innovation elements, constantly absorbing, integrating, and allocating various resources and innovating the incubation service mode, an important path to improving the performance of innovation incubation. Based on resource-based theory, network theory, and value chain theory, we proposed the conceptual model and research hypothesis for the relationship between innovation elements, incubation capacity, and innovation incubation performance in biomedical incubation platforms, with customized service as a moderating variable. The empirical results show that innovation elements have a significant positive impact on the improvement and transition of incubation capacity. Incubation capacity has a significant positive impact on innovation incubation performance in biomedical incubation platforms. Customized service plays a significant positive regulatory role between incubation capacity and innovation incubation performance in biomedical incubation platforms.

## Introduction

The biomedical industry is a typical innovation-driven industry and the development and application of new products and technologies play a key role in the market competitiveness of enterprises. Biomedical incubation platforms incubate incubatees by providing a variety of entrepreneurial supports, including physical space, consulting services, financial support, and network connections (1), thereby helping them to better access the value-based resources necessary for survival and development and promoting the success rate of innovation and entrepreneurship. A biomedical incubation platform as a professional incubator can provide professional startup facilities, equipment, and technical services for startups. It is currently one of the main development directions for incubators aiming to achieve specialization. Biomedical incubation platforms have fallen into “core rigidity” and “capacity trap,” along with frequent innovation iterations, intergenerational differences in incubation service functions, and diversified incubation needs. As a result, incubatees achieve little success in survival and development, capacity building, and incubation performance improvement. Innovation incubation aims to provide incubatees with intangible and higher value-added business service support (2). It seeks to guide the reconfiguration of incubation role functions and incubation links with industry chain construction as the main axis through various specialized strategies such as sustainable development, collaborative innovation, and technology transfer. The focus of biomedical incubation platforms on innovation elements has great practical significance for strengthening incubation capacity, improving incubation functions, and enhancing incubation performance.

Guided by resource-based theory, transaction cost theory, and social exchange theory, incubation platform research has focused on basic management functions such as basic services, performance evaluation, capacity enhancement, and service innovation as a resource-mediated broker. It has explored the impact mechanism of incubator performance enhancement at the micro level. Most researchers adopt the static perspective of various incubation target plans and incubation practice designs, ignoring the reality of resource mismatch in the innovation incubation process, whereby innovation elements are fragmented and scattered. Researchers have neglected the special characteristics derived from the incubation ecology of double embedding in positive externalities and the integration effect based on transaction costs and relationship values (3). Schwartz and Hornych (4) show that an industry focus can help incubation platforms to establish more specialized incubation management rules, create good synergies, and achieve an effective transfer of active knowledge and competitiveness to incubatees through peer-to-peer resource matching services, thereby improving incubation performance. Bruneel et al. (5) shows that the function and chronology of services provided by incubation platforms lead to generational differences and to differences in incubation functions and directions. According to strategic management theory, focusing on one or a few narrow market segments can achieve a sustainable competitive advantage. Incubation platforms obtain long-term incubation effects through the continuous strengthening of incubation functions such as industry focus, market focus, and network focus (6). This causes innovation incubation platforms to focus on the rational arrangement and design of innovation elements and promotes the clustering effect of multiple factors such as “government, industry, academia, research, users, and finance” to achieve increased innovation capacity (7). Therefore, the exploration of innovation incubation performance should be generalized by the intergenerational evolution of incubation platforms and categorized according to the different types of incubation platforms, value propositions, and incubation service functions.

Researchers have explored in-depth the mechanism of the influence of absorptive capacity, social capital, incubation platform control, network plurality, and innovation incubation performance from the perspective of power-change theory. However, most studies have explored the causal mechanism or boundary conditions of innovation incubation performance from a single perspective, ignoring the knowledge sourcing of innovation factor flow and allocation in the process of innovation incubation and the reality of organic integration and high synergy between the incubation platform's incubation capacity and external value-based resources. Therefore, this article constructs a conceptual model including innovation elements, incubation capacity, and innovation incubation performance of biomedical incubation platforms. It explores the mediating role of incubation capacity in the relationship between innovation elements and innovation incubation performance by using a structural equation model and examining the moderating role of customized services in the relationship between the incubation capacity and the innovation incubation performance of biomedical incubation platforms by using hierarchical regression analysis. This article aims to reveal the influence mechanism and the effectiveness mechanism of innovation factor focus in promoting advances in incubation capacity and enhancing the innovation incubation performance of biomedical incubation platforms. It aims to provide theoretical support and practical guidance for biomedical incubation platforms to enhance incubation performance and accelerate the growth of incubatees.

## Concept Definition and Study Hypothesis

### Innovation Elements

The composition of innovation factors mainly includes a simple two-dimensional classification taken from the direct factors of technology, capital, and human capital and the indirect factors of infrastructure, social environment, and macropolicies from the production factor perspective (8). It includes the main elements covering universities, institutions, and enterprises; resource elements such as information, knowledge, talents, and capital; and environmental elements such as internal hardware innovation environment and the external network innovation environment from a system and environment perspective (9). It also covers the three-dimensional structural elements of subject, support, and market (10) from the structure and function perspective. Integrating the above study foundation, the essential characteristics of biomedical incubation platforms, and the resource allocation characteristics of China's digital transformation context, we examined the connotation of innovation elements from the perspective of their roots and their essential factors. We concluded that innovation knowledge sources (11), incubation network environment (12), and value chain information (13) are the core elements that meet the characteristics of biomedical incubation platform and industry needs.

#### Innovation Knowledge Source

Innovation knowledge source is an important innovation element for science and technology enterprises. The high-tech characteristics of biomedical enterprises provide innovation knowledge source with a technological connotation. This connotation includes high-tech, scientific, and technological leaders, innovative creative thinking, and high-tech advanced medical equipment or instruments. The two main ways to obtain or contact innovative knowledge sources are to acquire external knowledge sources directly and to collaborate with organizations or individuals who have innovative knowledge sources. Zhang and Liu (14) explored the relationship between external innovation knowledge sources and absorptive capacity and breakthrough innovation performance and how fully utilizing external innovation knowledge resources can enhance absorptive capacity and innovation performance (15). Hou et al. (16) pointed out that external knowledge sources can achieve knowledge accumulation and update knowledge stock by continuously creating new knowledge through external cooperation, thereby effectively enhancing innovation performance (14). Incubated biomedical companies often find it difficult to achieve breakthrough innovation due to the lack of key technologies, scientific and technological talents, and backward medical equipment or instruments, forcing incubated biomedical companies to acquire knowledge of medical science and technology innovation. The acquisition of innovative knowledge sources, such as technical reports, technology transfer, patent databases, university–enterprise cooperation, and cooperation with third-party research institutions, can promote the incubation capacity of biomedical incubation platforms and enhance technological innovation. In particular, scientific and technological innovation talents and patents are regarded as the most valuable innovative knowledge sources at the knowledge and technology levels. This is of great significance to the overall construction of biomedical incubation platforms. Therefore, the following hypotheses are proposed.

H1: There is a significant positive correlation between innovative knowledge sources and the incubation capacity of biomedical incubation platforms.

#### Incubation Network Environment

The biomedical incubation platform incubation network refers to the organization beyond the nodes constituted by the biomedical incubation platforms through building intra-organizational and cross-organizational cooperation interfaces (16). These interfaces are embedded in the socioeconomic environment. They are linked to other incubators, governments, markets, suppliers, financial institutions, universities, research institutes, and other behavioral nodes, whose networking function can effectively overcome the difficulties of resource element scarcity, reducing the costs of resource search negotiation (17). He et al. (18) argue that an organization's network is regarded as a relationship of mutually beneficial and interdependent organizational forms, in which suppliers or partners provide resources to jointly design or jointly produce innovative services and products (19). The incubation network environment is specifically subdivided into government policy support, external capital investment, organizational and cultural atmosphere, infrastructure environment, and incubation network scale. Sun and Li (20) point out that network size reflects the richness of resource elements and that the growth of network size will prompt the incubation platform to improve its self-incubation capacity, thereby enhancing the market discourse and innovation incubation performance of the incubation platform (18). Zhao et al. argue that incubation platform networks break the traditional organizational boundaries and geographical restrictions by building new information service platforms and various incubation public service platforms to help incubatees integrate various innovation resources and realize the integration and distribution of incubation resources, thereby improving incubation performance (20). Gao et al. argue that incubation platforms provide incubatees with an effective mechanism to create, complement, and eventually commercialize proprietary knowledge through customer interface (supply side of innovation) and market interface (demand side of innovation) activities, connecting and leveraging international networks of knowledge creation and knowledge application, and breaking the boundaries of interorganizational and international knowledge flows (21). Biomedical incubation platforms focus on building an incubation network environment that is conducive to the co-evolution of themselves and their incubatees and to the improvement of innovation incubation performance. Therefore, the following hypothesis is proposed:

H2: There is a significant positive correlation between the incubation network environment of biomedical incubation platforms and incubation capacity.

#### Value Chain Information

The value chain information of biomedical incubation platforms refers to the information resources attached to the value chain activities, the main ways in which biomedical incubation platforms and incubatees break through organizational boundary restrictions to obtain significant external information and knowledge resources. Value chains provide an effective way for enterprises to acquire new knowledge and technology, to extract core knowledge and key technologies through organizational learning, to form strategic alliances, and to engage in other means of enhancing information and innovation capabilities (22). The amount of information provided by customers or suppliers through working relationships can increase the effectiveness of conscious and deliberate learning mechanisms (23). Lau and Lo argue that value chain information is the main way for firms to obtain information about innovation that contributes to their resource and knowledge base (24). Jiang and Liu (25) pointed out that value chain information has different source paths and that incubatees need to resource patchwork value chain information from divergent sources through exploratory learning to make it a directly usable isomorphic resource. Horizontal and vertical cooperation in the value chain can provide technical knowledge and market resources for firms, thereby enhancing their service capabilities and integrated functions (25). Biomedical incubation platforms use their own dual learning to form isomorphic resources from different sources of value chain information through decoding, encoding, transferring, and innovating to embed the incubation processes directly. Therefore, the following hypothesis is proposed:

H3: There is a significant positive correlation between value chain information and the incubation capacity of biomedical incubation platforms.

### Incubation Capacity

Incubation capability is the core competitiveness of a biomedical incubation platform. It consists of incubatees' self-incubation capabilities, which is the reflection of the comprehensive service capability of a biomedical incubation platform to incubate enterprises and its own growth capability (26). Colombo and Delmastro (27) showed that the capabilities of incubation platforms, such as talent pool, incubation experience, collaborative activities, innovation activities, and financial support, have a significant impact on the entrepreneurial performance of incubatees (28). Tian et al. (29) concluded that the innovation capabilities of incubation platforms facilitate the integration and utilization of existing technological know-how by incubatees and enhances incubation performance (30). Jiang and Tang (31) showed that there is a significant positive effect between the dynamic learning capacity of incubation platforms and innovation incubation performance (32). Jiang et al. argue that incubation platforms use network orchestration capabilities to enable the development of value platforms and subsequently improve innovation incubation performance (27). Wang et al. (33) showed that incubation capabilities can help incubatees to make better use of newly acquired knowledge and facilitate effective decision-making based on acquired new knowledge (29). Therefore, the following hypothesis is proposed:

H4: There is a significant positive correlation between incubation capacity and innovation incubation performance of biomedical incubation platforms.

### Moderating Effect of Customized Services

The term customized service means that the incubation platform provides peer-to-peer professional incubation services in response to the actual conditions and heterogeneous needs of incubatees at the growth stage (31). Service customization plays the role of high-quality endorser and driver, providing customized services. By “bundling-related resources,” it can achieve excellent customer value and reduce resource costs (34). In the context of the platform economy, biomedical incubation platforms continuously allocate priority resources to provide customized services to incubatees. They connect with external entities to carry out integrated customized services to meet the different needs of incubatees. This improves the incubation capacity of biomedical incubation platforms and accelerate the growth of incubatees, thereby enhancing the innovation incubation performance of biomedical incubation platforms (33). Zhang and Xiang (35) argued that customized knowledge-based services help supply and demand subjects form an interactive innovation interface, enhance the trust mechanism between collaborators, improve innovation capacity and service performance, and realize the dual value co-creation of system coupling and process coupling in the process of providing customized services in the areas of sales, consulting, alliance, and assistance (36). Biomedical incubation platforms provide peer-to-peer specialized services for incubatees in the complex situation of policy crossover and environment, rapidly improving incubation capacity and incubation quality. Therefore, the following hypothesis is proposed:

H5: Customized services have a significant positive moderating effect between the incubation capacity and innovation incubation performance of biomedical incubation platforms.

**Figure 1:**
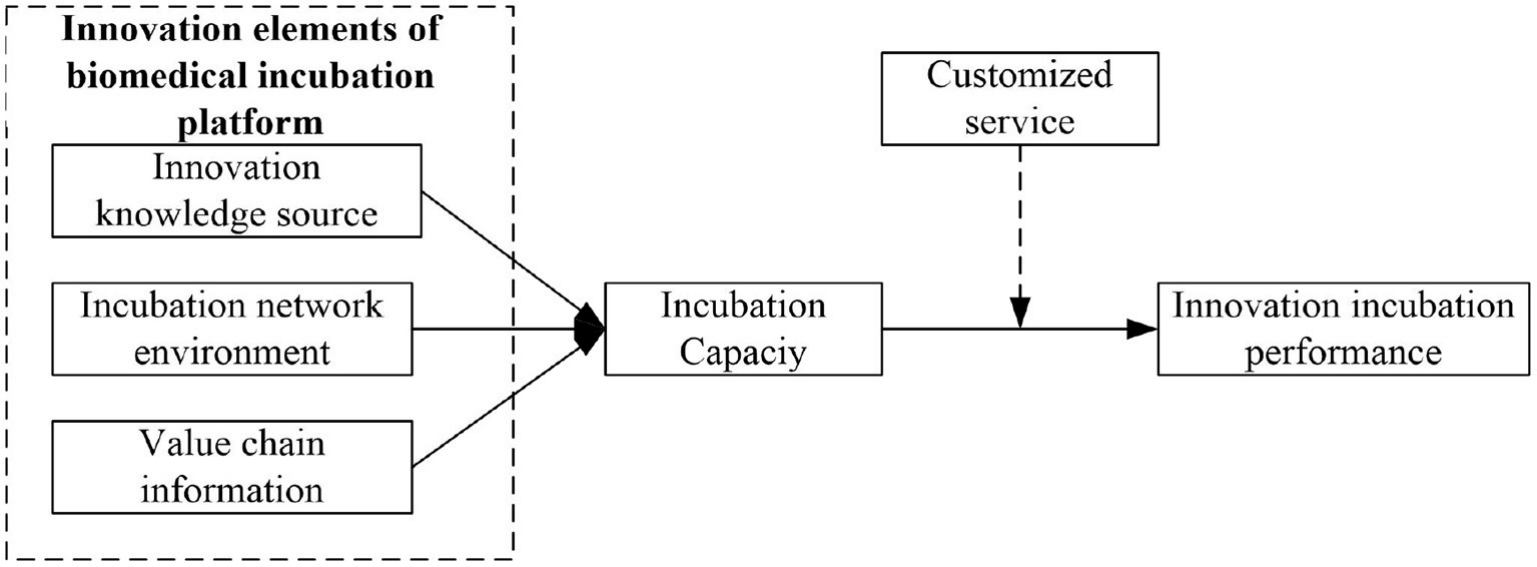
Conceptual model.

Figure 1 shows the conceptual model of this article.

## Research Methodology

### Data Collection

In this article, we study the influence of innovation elements and incubation capacity on the innovation incubation performance of biomedical incubation platforms. The sample is selected from incubation platforms such as biomedical industrial parks, incubators, and crowdsourcing spaces at or above provincial level as the survey objects. The Coronavirus disease 2019 (COVID-19) pandemic affected data collection. The data were obtained mainly through online questionnaires and video conference interviews with more than 10 regionally representative biomedical incubation platforms. The quality of data completion was ensured by arranging that the questionnaires were filled out anonymously by incubatee executives who were connected to the incubators and had a good grasp of the companies' innovation incubation. A declaration was made that the survey was intended for academic study and an undertaking was given promising the confidentiality of the questionnaires. From June to October 2021, 500 study questionnaires were distributed through email, WeChat, and other communication methods. A total of 358 questionnaires were collected, of which 38 questionnaires were invalid, leaving 320 valid questionnaires.

### Variable Measurement

The variables in this study were measured on a five-point Likert scale, with 1–5 indicating “totally disagree” to “totally agree.” The operationalized definitions and measurements of each variable were developed, designed, and improved based on relevant previous results.

Drawing on the study results of Zhou et al. (37) and Jiang et al. (25, 38), four scales were used to measure innovation knowledge sources: (i) acquiring high technology by incubation platform, (ii) signing up technology leaders by incubation platform, (iii) acquiring innovative creative thinking, and (iv) acquiring other innovative knowledge by incubation platform.

Drawing on the study results of Li et al. (39), four scales are used to measure the incubation network environment: (i) incubation platforms receive government policy support, (ii) incubation platforms improve organizational culture, (iii) incubation platforms enhance the infrastructure environment, and (iv) incubation platforms expand the incubation network scale.

Drawing on the study results of Guo et al. (13), Lau and Lo, (23), and Liu (24), and others, four scales are used to measure the value chain information: (i) the incubation platform gets technical information resources, (ii) the incubation platform gets market information resources, (iii) the incubation platform gets innovation information resources, and (iv) the incubation platform gets other information resources.

Drawing on the study results of Zhao et al. (35) and Sun and Li (40), four scales are used to measure the incubation capacity: (i) incubation platform's improved ability to cultivate incubatees, (ii) incubation platform's improved ability to provide comprehensive services to incubatees, (iii) incubation platform's improved ability to self-incubate, and (iv) incubation platform's enhancement of strategic awareness.

Drawing on the study results of Jiang et al. (27), four scales are used to measure customized services: (i) providing heterogeneous incubation services for incubatees based on their own resources, (ii) using relationship network resources to provide heterogeneous incubation services for incubatees, (iii) providing a docking place for incubatees and the two parties that can provide heterogeneous incubation services for them, and (iv) providing heterogeneous incubation services for incubatees in other ways that provide heterogeneous incubation services for incubatees.

Drawing on the study results of Tang et al. (41), four scales were used to measure the innovation incubation performance: (i) good growth of incubated enterprises, (ii) improvement of incubated enterprises' technological innovation capacity, (iii) good transformation of incubated enterprises' technological achievements, and (iv) significant improvement of incubated enterprises' graduation rate.

## Data Analysis and Results

### Descriptive Statistical Analysis

Table 1 lists the mean, SD, skewness, kurtosis, and the Pearson's correlation coefficient of innovation knowledge source, incubation network environment, value chain information, incubation capacity, customized service, and innovation incubation performance. The formal sample results in Table 3 show that the means for each question item range from 3.6141 to 3.7430 and the SDs range from 0.9732 to 1.1270. The skewness ranges from −1.101 to −0.546 and the kurtosis ranges from −0.790 to 1.072. The skewness and kurtosis satisfy the conditions of normal distribution, indicating that these 25 questions obey normal distribution. The correlation coefficients of innovation knowledge source, incubation network environment, and value chain information with incubation capacity are 0.452, 0.458, and 0.443, respectively. The correlation coefficients of incubation capacity and customized services are 0.452. The correlation coefficient of customized services and innovation incubation performance is 0.344. They are all significantly positively correlated at the 0.01 level. The correlation coefficients are all <0.7; there is no covariance. The data recovered from the questionnaire can be directly used in statistical analysis tests such as reliability analysis and structural equation model testing.

**Table 1:** Summary table of descriptive statistics.

**Variables**	**M**	**SD**	**Skewness**	**Kurtosis**	**1**	**2**	**3**	**4**	**5**	**6**
Innovation knowledge sources	3.6141	1.0869	−0.546	−0.79	1					
Incubation network environment	3.7430	0.9732	−1.050	1.072	0.565^**^	1				
Value chain information	3.6406	1.1270	−1.101	0.449	0.513^**^	0.484^*^	1			
Incubation capacity	3.6430	1.1179	−0.938	0.137	0.452^**^	0.458^**^	0.443^**^	1		
Customized services	3.6508	1.0812	−1.081	0.457	0.111^**^	0.107^**^	0.121^**^	0.452^**^	1	
Innovation Incubation Performance	3.6273	1.0527	−0.650	0.306	0.343^**^	0.288^**^	0.344^**^	0.337^**^	0.344^**^	1

* *Denotes p < 0.05*;

** *Denotes p < 0.01*.

### Reliability and Validity

In this study, the reliability and validity of the sample data were verified using Cronbach's α coefficients, combined reliability (CR), and average variance extracted (AVE) to ensure the authenticity and reliability of data analysis results. Table 2 presents the results of the reliability and validity tests for each variable. The Cronbach's α coefficients for innovation knowledge source, incubation network environment, value chain information, incubation capacity, customized services, and innovation incubation performance are 0.864, 0.895, 0.897, 0.924, 0.899, and 0.953. They are all >0.7. The CR is >0.8 and <1. The AVE is >0.5. The results indicate that all the dimensions of the study questionnaire have good reliability and validity.

**Table 2:** Reliability and validity analysis results.

**Variable**	**Number of**	**Cronbach's**	**CR**	**AVE**
**Variable**	**items**	**alpha**	**CR**	**AVE**
Innovation knowledge source	4	0.864	0.850	0.587
Incubation network environment	4	0.895	0.877	0.641
Value chain information	4	0.897	0.883	0.654
Incubation capacity	4	0.924	0.887	0.663
Customized services	4	0.899	0.908	0.712
Innovation incubation performance	4	0.953	0.942	0.803

*CR >0.6, AVE >0.5*.

### Structural Equation Modeling Analysis

Using Amos version 23.0 to perform structural equation modeling (SEM) analysis, as shown in Table 3, CMIN/DF is 1.952, which is between the criteria of 1–3. GFI and CFI are greater than the discriminant criteria of 0.9. RMSEA is 0.055, which is <0.08. AGFI is 0.888, which is >0.8 and slightly <0.9, in line with the discriminant acceptance range. Therefore, the SEM model can be considered to have a good fit.

**Table 3:** Structural equation model fit metrics.

**Fitting index**	**Acceptable range**	**Measured value**
CMIN		318.202
DF		163
CMIN/DF	1–3	1.952
GFI	>0.8, acceptable >0.9, good fit	0.913
AGFI	>0.8, acceptable >0.9, good fit	0.888
CFI	>0.9	0.969
RMSEA	<0.08	0.055

Table 4 shows that innovative knowledge sources have a significant positive effect on incubation capacity (β = 0.302, *p* < 0.01), indicating that the richer the innovation knowledge sources, the stronger the incubation capacity, thereby verifying hypothesis 1; incubation network environment has a significant positive effect on incubation capacity (β = 0.256, *p* < 0.01), indicating that the better the incubation network environment, the stronger the incubation capacity, thereby verifying hypothesis 2; value chain information has a significant positive effect on incubation capacity (β = 0.249, *p* < 0.001), indicating that the more value chain information is available, the stronger the incubation capacity, thereby verifying hypothesis 3; incubation capacity has a significant positive effect on innovation incubation performance (β = 0.322, *p* < 0.001), indicating that the stronger the incubation capacity of the biomedical incubation platform, the more significant the innovation incubation performance, thereby verifying hypothesis 4.

**Table 4:** Path coefficients.

**Path relation**			**Standardization factor**	**SE**	**CR**	**P**
Incubation capacity	←	Innovative knowledge source	0.302	0.097	3.115	0.002
Incubation capacity	←	Incubation network environment	0.256	0.089	2.890	0.004
Incubation capacity	←	Value chain information	0.249	0.072	3.473	***
Innovation incubation performance	←	Incubation capacity	0.322	0.052	6.241	***

### Test for Moderating Effect of Customized Services

Three-step hierarchical regression analysis sets the independent variable as incubation capacity; the dependent variable is set as innovation incubation performance and the interaction term of “incubation capacity × customized service” is added. Table 5 shows the results of the moderating effect test, R2 is 0.113, 0.159, and 0.169 and the regression coefficients are significant. Therefore, customization services have a positive moderating effect on the effect of incubation capacity on innovation incubation performance, thereby verifying hypothesis 5.

**Table 5:** Moderating effect of customization services.

	**Innovation incubation performance**		
	**Step 1**	**Step 2**	**Step 3**
**Independent variables**			
Incubation capacity	0.317***	0.214***	0.245***
**Adjustment variables**			
Customized services		0.231***	0.260***
**Interaction items**			
Incubation capacity*Customization services			0.067***
R2	0.113	0.159	0.169
ΔR2	0.113	0.046	0.009
ΔF	40.636***	17.394***	3.550***

^*^Denotes p < 0.05; ^**^Denotes p < 0.01; Denotes p < 0.001.

## Conclusion and Implications

In the innovation incubation process of biomedical incubation platforms, innovation elements (innovation knowledge source, incubation network environment, and value chain information) have a significant positive impact on the innovation incubation performance of biomedical incubation platforms through the mediating role of incubation capacity. Customized services play a significant moderating role between incubation capacity and innovation incubation performance of biomedical incubation platforms. All five hypotheses were verified. The findings of this article have good theoretical reference significance and practical value for the focus of biomedical incubation platforms on the multiple innovation elements and their effective allocation in the incubation process and the improvement of innovation incubation performance of biomedical incubation platforms.

(1) Innovation elements (innovation knowledge source, incubation network environment, and value chain information) have a significant positive impact on incubation capacity. Incubation capacity as a mediating variable has a significant positive impact on the innovation incubation performance of biomedical incubation platforms. Biomedical incubation platforms directly improve innovation incubation performance by focusing on innovation elements in their operation and management practices. The focus of three innovation elements positively contributes to the survival rate and the growth status of incubatees, the cultivation of incubatees' scientific and technological innovation capabilities, the transformation of scientific and technological achievements, and the graduation rate of incubatees.

First, social capital, good entrepreneurial opportunities, and highly cooperative teams were viewed as the most important factors for entrepreneurship in China (42). Biomedical incubation platforms enhance innovation incubation performance by focusing on external sources of innovation knowledge such as focusing on biomedical R&D and innovation with organizations or individuals with source knowledge such as universities, key research institutes, key state laboratories, high-tech enterprises, advanced equipment manufacturing enterprises, and leading scientific and technological talents in the form of direct reading or collaborative cooperation and innovation, to continuously improve incubation capacity and to enhance innovation incubation performance.

Second, biomedical incubation platforms grasp the development direction of innovation and entrepreneurship policies, focus on building an ecosystem platform for an incubation network, and build broad open-source incubation platforms by creating a harmonious organizational and cultural atmosphere for biomedical incubation platforms, incubatees, and multiple external subjects. These actions will expand the scale of the incubation network and will attract capital and technology from external subjects to create good incubation platforms. Technology and other factors create a good resource environment for the incubation network, continuously improving the incubation capacity and improving innovation incubation performance.

Third, biomedical incubation platforms and incubatees obtain a large number of external information resources through the value chain to help them to break through the organizational boundary restrictions in business management practices and use diverse information resources such as technical information, market information, innovation information, and other kinds of information resources to respond appropriately to the challenges brought by fierce market competition and continuous development. Therefore, during the operation and management of biomedical incubation platforms, managers should strengthen the strategic awareness of innovation element acquisition and continuously strengthen the importance of the innovation element in biomedical incubation platform incubation capacity improvement. This would greatly enhance the incubation capacity and the comprehensive service capacity of incubated enterprises. It would also improve biomedical incubation platforms' own incubation capacity and promote decision-makers' management and innovation resources to compete for strategic awareness. Improvements in these aspects are essentially incubation capacity improvements.

(2) Customized services have a significant moderating effect between incubation capacity and innovation incubation performance. The higher level of customization services in the biomedical incubation platform is conducive to improving the range of incubation capacity, implying a greater contribution to innovation incubation performance. With high incubation capacity, a biomedical incubation platform can provide customized services to incubatees according to their growth stages and heterogeneous needs, thereby regulating the relationship between incubation capacity and innovation incubation performance and realizing the path of innovation incubation performance improvement mediated by incubation capacity. Customized services, as a moderating variable between incubation capacity and innovation incubation performance, work in two main ways. First, the biomedical incubation platform uses implicit knowledge and its a priori resource advantages to the full to provide order-based incubation services to meet the real needs of incubatees. Second, the biomedical incubation platform builds an incubation network ecosystem platform with external network subjects through customer interface, market interface, and technology interface activities, attracting multiple subjects to participate in solving incubatees' incubation problems with the incubation platform, motivating incubation parties to use the incubation platform as an interface to achieve knowledge interaction, and realize customized needs and services.

## Data Availability Statement

The original contributions presented in the study are included in the article/supplementary material, further inquiries can be directed to the corresponding author/s.

## Ethics Statement

Approved by the Scientific Research Office of Sanya University, this article named Innovation Elements, Incubation Capacity, and Incubation Performance in Biomedical Incubation Platform: Moderating Role of Customized Services, written by Jiang Qian's research group, aims to illustrate the internal mechanism and effect of how innovation elements, incubation capability, and other variables affect the performance of innovation incubation on biomedical incubation platforms. The research purpose, research plan, research method, research content, and implementation process of this study all meet the relevant ethical requirements of Sanya University. We give our permission for future studies based on our study and please ensure that any follow-up study is in strict accordance with our study plan.

## Author Contributions

QJ: paper design, data analysis, and article writing. DW: paper design and data collection. YFW: paper design and article instruction. BYW: data analysis. All authors contributed to the article and approved the submitted version.

## Conflict of Interest

The authors declare that the research was conducted in the absence of any commercial or financial relationships that could be construed as a potential conflict of interest.

## Publisher's Note

All claims expressed in this article are solely those of the authors and do not necessarily represent those of their affiliated organizations, or those of the publisher, the editors and the reviewers. Any product that may be evaluated in this article, or claim that may be made by its manufacturer, is not guaranteed or endorsed by the publisher.
